# Exploring Tissue- and Sex-Specific DNA Methylation in Cattle Using a Pan-Mammalian Infinium Array

**DOI:** 10.3390/ijms26094284

**Published:** 2025-05-01

**Authors:** Zhenbin Hu, Clarissa Boschiero, Mahesh Neupane, Nayan Bhowmik, Liu Yang, Levi Kilian, James Mel DeJarnette, Mehdi Sargolzaei, Bo Harstine, Cong-Jun Li, Wenbin Tuo, Ransom L. Baldwin, Curtis P. Van Tassell, Charles G. Sattler, George E. Liu

**Affiliations:** 1 Animal Genomics and Improvement Laboratory, Beltsville Agricultural Research Center, Agricultural Research Service, United States Department of Agriculture, Beltsville, MD 20705, USA; 2 Select Sires Inc., 11740 U.S. 42 North, Plain City, OH 43064, USA; 3 Animal Parasitic Diseases Laboratory, Beltsville Agricultural Research Center, Agricultural Research Service, United States Department of Agriculture, Beltsville, MD 20705, USA

**Keywords:** cattle, DNA methylation, tissue-specific DNA methylation, sex-specific DNA methylation, HorvathMammalMethyl-Chip40

## Abstract

DNA methylation is crucial in gene expression regulation and tissue differentiation in livestock. However, genome-wide methylation patterns among tissues remain underexplored in cattle, one of the world’s most important farm animals. This study investigates sex- and tissue-specific DNA methylation in cattle using CpG site methylation data generated by an Infinium DNA Methylation array (HorvathMammalMethyl-Chip40) across seven tissues. Our analysis revealed significant tissue-specific methylation differences, with reproductive tissues/cells, such as the sperm, exhibiting distinct profiles compared to somatic tissues like hair and blood. Principal component analysis (PCA) highlighted tissue differentiation as the primary driver of methylation variability. We also identified 222 CpG sites with significant sex-based methylation differences, particularly on the X chromosome, suggesting the potential epigenetic regulation of sex-specific traits. The Gene Ontology (GO) enrichment analysis indicated that these methylation patterns may influence biological processes such as epithelial cell proliferation and blood vessel remodeling. Overall, this study provides important insights into sex- and tissue-specific epigenetic regulation in cattle, with implications for improving livestock breeding strategies through integrating epigenetic data.

## 1. Introduction

Epigenetic modifications, particularly DNA methylation, are essential in regulating gene expression and play pivotal roles in tissue differentiation, development, and maintaining cellular identity in humans and animals [1]. DNA methylation, especially at CpG dinucleotides, involves the addition of a methyl group to cytosine residues. This modification typically leads to transcriptional repression by altering chromatin structure or inhibiting the binding of transcription factors [2,3]. It is crucial for processes such as embryonic development, immune system function, and metabolic regulation in mammals, including cattle. Understanding tissue-specific methylation patterns in livestock can provide valuable insights into the molecular mechanisms behind tissue differentiation and economically important traits. These traits include milk production, growth rate, and disease resistance [4,5,6,7]. The advent of high-throughput array and sequencing technologies has revolutionized the field of epigenetics, enabling a comprehensive analysis of DNA methylation across different tissues and developmental stages. Tools such as the Infinium DNA Methylation array have facilitated the exploration of conserved CpG sites in humans and mice, allowing researchers to investigate methylation patterns across various tissues. One of the significant recent developments in this field is the creation of a pan-mammalian DNA methylation array, HorvathMammalMethyl-Chip40, pioneered by Horvath and colleagues [8,9]. Designed to work across mammals, including cattle, this array provides a powerful method for identifying tissue-specific and age-related methylation changes across mammals. Leveraging this technology enables researchers to gain novel insights into how DNA methylation regulates gene expression and influences phenotypic traits in diverse biological contexts.

While much of the research on DNA methylation has focused on humans and model organisms, there is growing recognition of the importance of studying epigenetic modifications in other mammals [9]. Cattle, for example, play a vital role in global food security. Traits such as milk yield, feed efficiency, and disease resistance are critical for improving livestock productivity. Epigenetic regulation, including DNA methylation, has emerged as a critical factor influencing these traits by modulating gene expression in a tissue-specific manner [10,11]. Previous studies in cattle have shown that methylation patterns in tissues such as blood, liver, and muscle can influence growth, reproductive performance, and immune responses [6,7,12,13,14]. However, comprehensive studies examining methylation across multiple tissues in cattle remain limited, particularly those utilizing high-throughput methods like the Infinium DNA Methylation array [15,16,17].

The current study aims to expand our understanding of DNA methylation in cattle by investigating methylation patterns across seven distinct tissues, including sperm, hair, and blood, using the HorvathMammalMethyl-Chip40 array. By examining CpG site methylation in these tissues, we seek to uncover the extent of tissue-specific methylation and identify key CpG sites that may regulate important physiological and developmental processes. Tissue-specific methylation patterns are crucial for maintaining the identity and function of different cell types. Dysregulation of these patterns can lead to various disorders or negatively affect animal performance traits. In addition to tissue-specific methylation, sex-based differences in DNA methylation have also been observed in multiple species, including cattle [18]. Such differences are often linked to regulating genes on the X chromosome or genes involved in reproductive and immune functions. Investigating these sex-based methylation patterns in cattle can reveal how epigenetic regulation contributes to sex-specific traits, such as reproductive performance and disease susceptibility [19]. Thus, this study also explores the potential impact of sex on methylation patterns across different tissues, providing deeper insights into epigenetic regulation in cattle.

## 2. Results

### 2.1. Application of the Infinium DNA Methylation Array in Cattle

**Figure 1:**
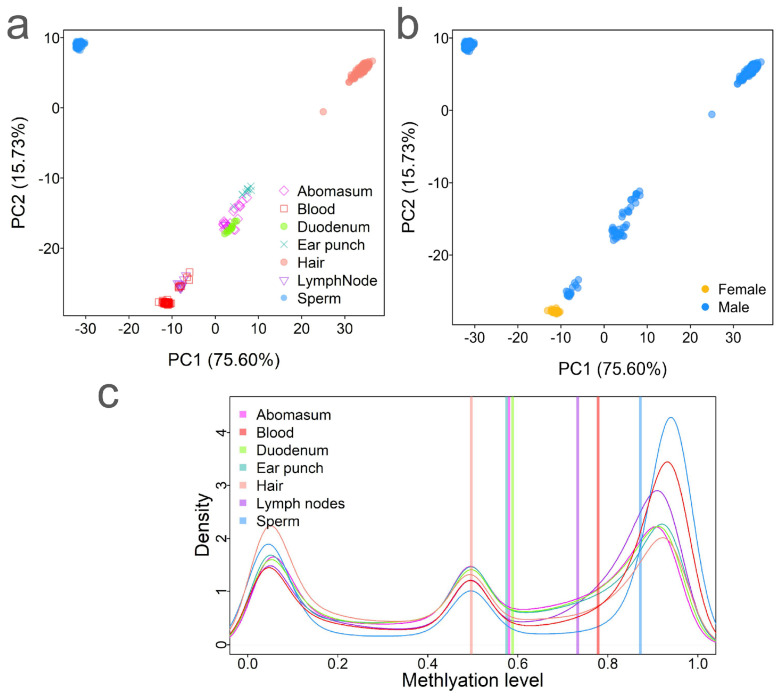
Methylation variations among tissues. (**a**) Principal component analysis (PCA) of DNA methylation illustrating the differences among 433 samples based on the tissue type. (**b**) PCA showing methylation differences by sex. (**c**) Comparison of CpG site methylation levels across tissues. Vertical bars represent the average methylation levels for each tissue type.

To explore the tissue specificity and differentiation of methylation among tissues in cattle, we integrated newly generated CpG methylation data for 162 sperm, 166 hair, 10 ear punch, and 7 blood samples, as well as the previously published data [20,21]. This resulted in a total of 433 methylation datasets across seven tissues or cell types, including 20 abomasum, 10 duodenum, 55 blood, 166 hair, 10 ear punch, 10 lymph node, and 162 sperm samples (Figure 1a). All the methylation data were generated using the HorvathMammalMethyl-Chip40 methylation array, which was designed based on conserved genomic regions across a wide range of mammalian species [8]. Methylation levels for 37,554 CpG sites (out of the original 38,608) were successfully quantified across the 433 samples (Figure 1a). Among these, 48 blood samples were collected from females and 385 samples were collected from males (Figure 1b). The number of samples for each tissue/cell type ranged from 10 (duodenum, ear punch, and lymph node) to 166 (hair) (Figure 1a).

### 2.2. Methylation Variations Among Tissues in Bos taurus

To assess methylation variations across tissues, we performed principal component analysis (PCA) on the CpG methylation data. The results revealed strong clustering by tissue/cell type and sex, indicating that these are the primary factors influencing methylation variability (Figure 1a,b). The first principal component (PC1, 75.60%) mainly captured the difference between the germline cells (sperm) and the somatic tissues. The second PC (PC2, 15.73%) reflected variation among tissues and between sexes (Figure 1b). We observed a distinct methylation pattern in blood samples between sexes (Figure 1a,b). The samples clustered mainly by tissue type, confirming that tissue/cell type differentiation is the major driver of methylation variation. From bottom to top, the clustering pattern followed the order of blood, lymph node, duodenum, abomasum, ear punch, and hair. Interestingly, sex-based methylation differences in blood were also observed (Figure 1a,b), suggesting a distinct methylation landscape between males and females, which aligns with the findings from human studies [22].

To further investigate global DNA methylation differences among tissues, we compared their overall methylation levels. Sperm exhibited the highest median methylation level (0.87), while hair had the lowest level (0.50) (Figure S1). The density distribution of methylation levels revealed three distinct peaks: low, intermediate, and high methylation (Figure 1c). Notably, sperm displayed the most polarized methylation distribution and the highest average methylation, consistent with observations from previous human and cattle studies [7,23]. In contrast, hair exhibited a relatively even distribution across low, intermediate, and high methylation levels (Figure 1c).

### 2.3. Methylation Relationships Among Tissues

**Figure 2:**
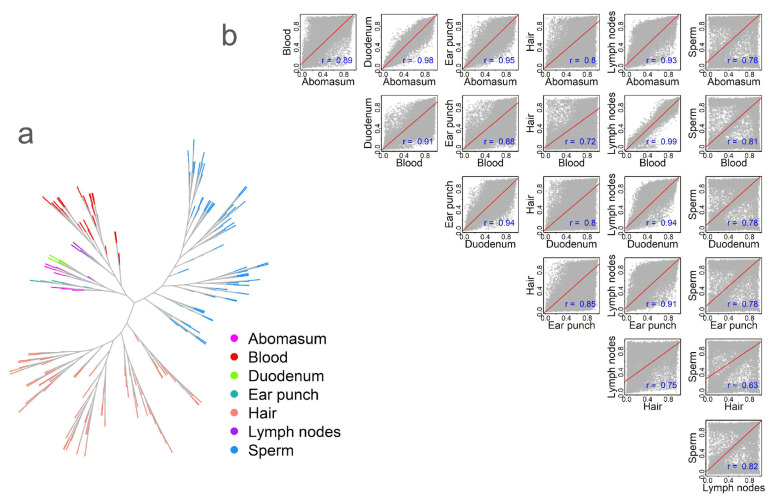
Tissue relationships based on DNA methylation. (**a**) Methylation-based phylogenetic tree, with different colors representing different tissue types. (**b**) Correlation of methylation levels across tissues, with correlation values labeled in blue. Correlation coefficients were calculated using Spearman’s correlation. All correlations were statistically significant at *p* < 0.01.

To analyze methylation similarity among tissues, we calculated the Spearman correlation of averaged methylation levels across tissues/cell types. First, we constructed a correlation matrix and visualized it using a phylogenetic tree-like method (Figure 2a). The results revealed a tissue-centric pattern, with variation within specific tissues represented as separate branches. This within-tissue variation may reflect natural biological variation [10,24]. Next, we calculated the methylation correlation across tissues based on the averaged methylation for each CpG site. Tissues belonging to the same physiological systems exhibited higher correlations (Figure 2b). For example, the strongest correlation (*r* = 0.99, *p* < 0.01) was observed between the blood and lymph nodes. In contrast, the lowest correlation (*r* = 0.63, *p* < 0.01) was found between hair and sperm (Figure 2b). Scatterplots further highlighted distinct methylation patterns. When comparing sperm to other tissues, we observed numerous CpG sites that were either highly hypermethylated or hypomethylated (Figure 2b). In contrast, comparisons involving hair revealed a greater number of hypomethylated CpG sites—falling below the regression line—than hypermethylated sites (Figure 2b).

### 2.4. CpG Methylation Patterns Are Highly Tissue-Specific

**Figure 3:**
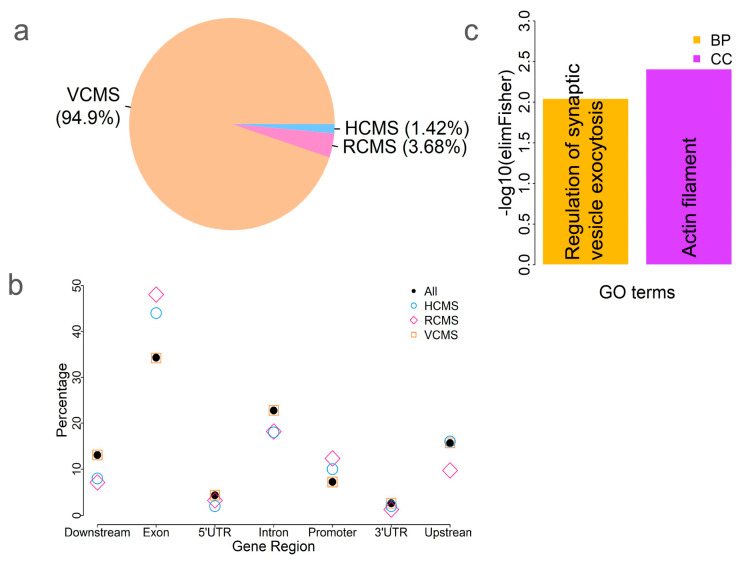
CpG site methylation variation among tissues. (**a**) Counts of three different types of CpG sites: highly conserved methylated CpG sites (HCMS, *p* > 0.05); relatively conserved methylated CpG sites (RCMS, *p* between 2.66 × 10^−7^ and 0.05), and variably conserved methylated CpG sites (VCMS, *p* < 2.66 × 10^−7^). (**b**) Comparison of these three types of CpG sites, as well as all CpG sites, relative to various genic features. (**c**) GO enrichment analysis of the 48 genes near or within HCMS. BP—biological process and CC—cellular component.

The above results showed that tissues clustered based on methylation variation (Figure 1 and Figure 2), indicating tissue-specific methylation patterns. Next, we explored which CpG sites were conserved versus variable among tissues. To identify tissue-specific CpG methylation (varied among tissues) and conserved CpG sites, we compared the methylation levels of each CpG site across tissues. We identified 532 highly conserved methylated CpG sites (HCMS) by comparing the differences among tissues (ANOVA, *p* > 0.05). These HCMS were located near or within 48 genes (Table S1). We also identified 1383 relatively conserved methylated CpG sites (RCMS), with p-values ranging from 2.66 × 10⁻^7^ (based on a Bonferroni threshold of 0.01/37,554, corresponding to the number of HorvathMammalMethylChip40 probes) to 0.05. CpG sites were classified as variably conserved methylated CpG sites (VCMS) if they had *p* < 2.66 × 10^−7^ (ANOVA). We identified 35,639 VCMS, accounting for 93.48% of all CpG sites, indicating that most CpG sites varied significantly among tissues (Figure 3a). To examine the genomic distribution of these three CpG site categories, we compared their occurrences across different genic features. Our results showed that HCMS and RCMS were enriched in the exon and promoter regions compared to the full set of CpG sites (Figure 3b), indicating that methylation is more conserved in these functional genomic regions among tissues.

To investigate the potential function of the conserved CpG sites, we performed a GO enrichment analysis of the 48 genes associated with HCMS (Table S1). The results showed that they were enriched in a cellular component GO term: actin filament (GO:0005884; *p* = 0.0039) and a biological process GO term: regulation of synaptic vesicle exocytosis (GO:2000300; *p* = 0.009) (Figure 3c). Actin filaments, also known as microfilaments, play roles in numerous cellular processes, including cell division, motility, morphology, and muscle contraction. Actin is highly conserved and participates in many protein–protein interactions [25]. The results imply the conservation of the gene functions regulated by methylation.

### 2.5. Identification of Tissue-Specific CpG Sites

**Figure 4:**
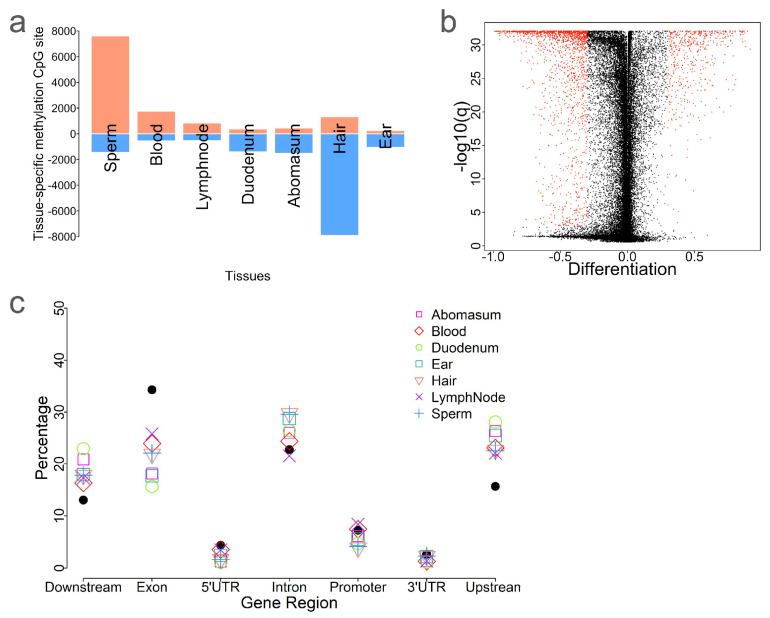
Tissue−specific differentially methylated CpG sites. (**a**) Identification of tissue-specific differentially methylated CpG sites (tDMS). Blue denotes hypomethylated CpG sites, while red denotes hypermethylated CpG sites. (**b**) Volcano plot of tDMS associated with blood, with red dots indicating significantly identified CpG sites. (**c**) Comparison of the distribution of tDMS across tissues and all CpG sites relative to genic features.

Next, we identified the tissue-specific differentially methylated CpG sites (tDMS) by comparing the methylation level of each CpG site in a given tissue against all other tissues. The number of tDMS ranged from 1300 in ear punches to 9227 in hair (Figure 4a and Table 1). Most tDMS in sperm (84.18%, 7609/9039), blood (76.46%, 1758/2305), and lymph nodes (61.73%, 839/1359) were hypermethylated (Figure 4a and Table 1). In contrast, most tDMS in hair (85.66%, 7904/9227), duodenum (79.26%, 1448/1761), and abomasum (77.81%, 1522/1956) were hypomethylated (Figure 4a and Table 1). To explore the distributions of these CpG sites, we compared the distribution of these tDMS to all CpG sites (Figure 4c). Interestingly, tDMS were enriched in intron and upstream/downstream regions of genes but were underrepresented in exonic regions. This suggests that exon methylation tends to be more conserved among tDMS.

We also performed a GO term enrichment analysis for the gene sets associated with tDMS in each tissue (Table S2). The number of enriched GO terms ranged from 26 (lymph nodes) to 46 (blood). In total, 126 GO terms were identified, most of which were associated with biological processes (Table S3). For sperm, 2290 sperm-tDMS-associated genes were enriched in 35 GO terms, including 28 related to biological process, 6 to molecular function, and 1 to cellular component (Table S3). Several transcription regulation terms were enriched, including GO:0045944 (positive regulation of transcription by RNA polymerase II), GO:0000122 (negative regulation of transcription by RNA polymerase II), GO:0001228 (DNA-binding transcription activator activity, RNA polymerase II-specific), and GO:0005667 (transcription regulator complex). Notably, several transcription regulation GO terms were repeatedly identified for different tissues, such as positive regulation of transcription by RNA polymerase II (GO:0045944), suggesting the role of DNA methylation in regulating tissue-specific gene expression (Table S3).

### 2.6. Sex Differences of DNA Methylation in Blood

**Figure 5:**
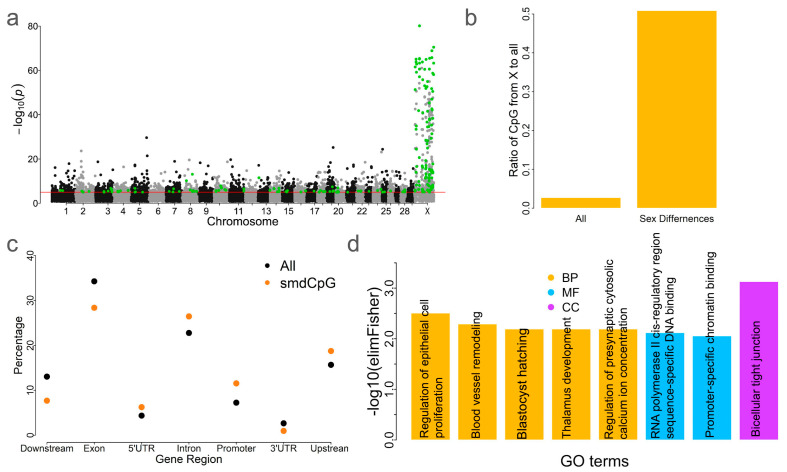
Methylation in blood differs between sexes. (**a**) Genome-wide distribution of sex-methylation-differentiated CpG sites (smdCpG). The red line indicates the genome-wide significance threshold and green dots represent significant smdCpG sites. (**b**) Enrichment analysis of smdCpG sites on the X chromosome. “All” indicates the proportion of all CpG sites on the X chromosome relative to all CpG sites used in this study, while “Sex Differences” represents the proportion of smdCpG sites on the X chromosome relative to all CpG sites on the X chromosome. (**c**) DistributionAll indicates the ratio of smdCpGall CpG sites from the X chromosome to all CpG sites used in this study, while sex differences mean the ratio of identified sex-different CpG sites on the X chromosome to all CpG sites on the X chromosome. (**c**) Comparison of the distribution of smdCpG sites and all CpG sites relative to genic features. (**d**) GO term enrichment analysis of smdCpG sites. BP—biological process, MF—molecular function, CC—cellular component.

Given the observed differences in blood methylation patterns between sexes (Figure 1b), we sought to identify sex-methylation-differentiated CpG sites (smdCpG). By comparing CpG methylation levels between males and females, we identified 222 smdCpG sites with a Q value < 0.0001 and an absolute methylation difference >0.3 (Figure 5a). These CpG sites were significantly enriched on the X chromosome (*n* = 113, *p* < 0.01, Chi-square test) (Figure 5b). Of the 222 smdCpG sites, 184 were hypermethylated in females. This trend is consistent with previous findings in humans [26]. Higher proportions of smdCpG sites, relative to all CpG sites, were found within intronic and promoter regions (Figure 5c). Notably, 115 of the 222 smdCpG sites (52%) overlapped with blood tDMS. These 222 CpG sites were associated with 122 genes that were enriched in 8 GO terms: i.e., 5 biological process, 2 molecular function, and 1 cellular component term(s) (Figure 5d). The most significantly enriched GO term based on the *p*-value was bicellular tight junction (GO:0005923, *p* = 0.00074) (Figure 5d). The top biological process GO term was regulation of epithelial cell proliferation (GO:0050678, *p* = 0.003). Previous studies have shown that sex influences intestinal epithelial stem cell proliferation [14], with males exhibiting lower epithelial cell proliferation rates than females [27]. The second most significant GO term was blood vessel remodeling (GO:0001974, *p* = 0.005). Additionally, blastocyst hatching (GO:0001835, *p* = 0.0064) was also enriched, consistent with prior reports of gene expression differences between male and female blastocysts [28].

## 3. Discussion

This study presents a comprehensive analysis of tissue-specific and sex-differentiated DNA methylation patterns in cattle utilizing the Infinium DNA methylation array. The identified tissue-specific CpG sites provide an epigenomics basis for tissue differentiation [29]. Our findings significantly enhance the current understanding of epigenetic regulation in cattle, revealing both tissue-specific and sex-based differences in methylation patterns. These results contribute to the growing knowledge of livestock epigenetics and offer potential applications for improving cattle genomics-enabled breeding strategies by integrating epigenetic data [30].

One of the major findings of this study is the strong tissue specificity of methylation patterns. Principal component analysis (PCA) demonstrated that tissue type is the primary driver of methylation variations (Figure 1). The result aligns with prior research on tissue-specific DNA methylation in mammals and model organisms [2,31]. Tissues such as sperm, hair, and blood exhibited distinct methylation profiles, with some enriched for specific CpG sites. For instance, sperm samples were notably enriched for hypermethylated CpG sites, while somatic tissues like hair exhibited a broader distribution of methylation levels. The methylation pattern may be related to specific gene expression patterns in sperm [32]. These findings suggest that epigenetic regulation through DNA methylation plays a critical role in maintaining tissue-specific functions, such as spermatogenesis in sperm or hair follicle development in the skin [19,33,34]. Identifying these tissue-specific CpG sites not only improves our understanding of tissue differentiation but also opens possibilities for identifying biomarkers linked to economically important traits in cattle, such as fertility or disease resistance.

Although blood was the primary tissue analyzed for sex-based methylation differences, the findings highlight the importance of considering sex as a variable in future epigenetic studies. They also underscore the value of exploring additional tissues or single-cell-based methylation differentiation between sexes in cattle [35]. The study identified a subset of CpG sites with significant sex-based differences in DNA methylation (smdCpG) (Figure 5). These smdCpG sites were predominantly located on the X chromosome, consistent with previous studies demonstrating the role of X chromosome inactivation and dosage compensation in regulating sex-specific traits [36,37]. However, some smdCpG sites were identified on autosomes. Sex-differentiated expressed genes were also identified on autosomes in humans [38], which indicated that autosome-harbored genes play important roles in shaping the difference between sexes.

The GO enrichment analysis revealed that smdCpG sites were enriched in biological processes such as epithelial cell proliferation (Figure 5c), suggesting their involvement of these pathways in physiological differences between sexes. Epithelial cell proliferation is associated with gonadal development and is regulated by sex hormones like testosterone (males) and estrogen (females), contributing to the distinct characteristics of male and female reproductive organs [39]. Future studies should aim to generate a comprehensive methylation differentiation map across multiple tissues to better understand the fine-scale landscape of sex-based methylation differences and integrate this information into cattle trait discovery and improvement.

The potential applications of these findings are vast. For example, identifying tissue-specific methylation patterns in reproductive tissues like sperm could inform the development of biomarkers for fertility. Fertility is a critical trait in dairy and beef cattle, and improving it is essential for the sustainability of livestock production. Methylation patterns that are stable across generations may serve as epigenetic markers that breeders could use to predict reproductive success or the likelihood of offspring inheriting specific traits. Similarly, DNA methylation markers in somatic tissues like blood could be used for early disease detection or as indicators of an animal’s health status, allowing for more informed management decisions.

Moreover, this study highlights the importance of using advanced genomic tools, such as the Infinium DNA Methylation array, to explore epigenetic variation. The array’s ability to capture methylation across thousands of CpG sites at high resolution makes it a valuable tool for livestock research. As genomic technologies continue to evolve, more integrative studies combining DNA methylation, gene expression, and phenotypic data will be critical to uncovering the complex interactions between genotype, epigenotype, and phenotype in livestock. Such studies will likely lead to the identification of novel regulatory mechanisms influencing economically important traits, offering new opportunities for genetic improvement programs.

Despite the significant contributions of this study, several limitations should be acknowledged. First, focusing on a limited number of tissues may not fully capture the breadth of epigenetic variation in cattle. Future studies should include additional tissues, such as muscle or adipose tissue, to provide a more comprehensive view of methylation patterns across the entire organism. Future studies should also expand sex-specific methylation analyses to a broader range of tissues to uncover potential tissue-dependent patterns and gain deeper insights into the regulatory mechanisms underlying sex differences in cattle. Second, while this study identified key tissue- and sex-specific CpG sites, the functional consequences of these methylation changes remain to be fully explored. Future integration of methylation data with gene expression, quantitative trait loci, and other datasets would provide a more complete understanding of the biological significance of these findings.

In conclusion, this study offers new insights into the epigenetic regulation of gene expression in cattle through the analysis of tissue-specific and sex-based DNA methylation patterns. Our findings contribute to the growing understanding of how DNA methylation shapes tissue differentiation and the sexes. These results have significant implications for improving cattle breeding strategies, particularly in the development of precision breeding approaches that incorporate epigenetic data. As livestock genomics advances, integrating DNA methylation into breeding programs holds promise for enhancing productivity, animal health, and sustainability. Incorporating epigenetic data into breeding strategies represents a promising step toward achieving precision agriculture and sustainable livestock production [40].

## 4. Materials and Methods

### 4.1. Methylation Data Generation

DNA was extracted from the sperm of 162 cattle and the hair of 151 cattle using the DNeasy Blood and Tissue Kit (Qiagen, Germantown, MD, USA). DNA concentrations were measured using Qubit (Thermo Fisher Scientific, Waltham, MA, USA). Samples were sent to the Clock Foundation at the Harbor-UCLA Medical Center (Torrance, CA, USA) for a DNA methylation analysis using the HorvathMammalMethyl-Chip40 array, which includes 37,492 CpG sites conserved across mammalian species [8]. The probes were aligned to the cattle genome assembly. Raw .idat files were processed using the minfi package [41] to generate normalized methylation levels, expressed on a scale from 0 (unmethylated) to 1 (fully methylated). CpG site methylation levels were normalized to β-values using the SeSAMe package [14].

### 4.2. Principal Component Analysis

Principal component analysis was performed using the prcomp function in R (version 4.3.1).

### 4.3. Phylogenetic Tree

A hierarchical clustering tree was constructed using the hclust function in R with a distance matrix. The tree was then converted into a phylogenetic format using the as.phylo function and visualized with the plot.phylo function in the ape package [42].

### 4.4. Methylation Correlation Between Tissues

For each CpG site, methylation values were averaged within each tissue. Pearson’s correlation was used to calculate the methylation similarity between tissues.

### 4.5. Identification of Highly Variable and Conserved Methylated CpG Sites

To assess methylation variability across tissues, we classified CpG sites into three categories based on ANOVA *p*-values: highly conserved methylated CpG sites (HCMS) with *p* > 0.05; relatively conserved methylated CpG sites (RCMS) with *p*-values between 2.66 × 10⁻^7^ (based on a Bonferroni threshold of 0.01/37,554, corresponding to the number of HorvathMammalMethylChip40 probes) and 0.05; and variably conserved methylated CpG sites (VCMS) with *p* < 2.66 × 10⁻^7^.

### 4.6. Tissue-Specific CpG Identification

Tissue-specific CpG sites were identified by comparing the methylation levels of each CpG site in a given tissue against all other tissues using the Wilcoxon signed-rank test, implemented with the wilcox.test function in R. The false discovery rate was controlled using the qvalue package (version 2.32.0). CpG sites with a *q*-value < 0.001 and an average methylation difference greater than 0.3 were considered tissue-specific.

### 4.7. GO Enrichment Analysis

The GO enrichment analysis was performed as previously described [21]. Briefly, CpG-associated genes were used as the background to reduce bias. Enrichment was conducted using the *TopGO* package with algorithm = “weight01” and statistic = “fisher”. GO terms with a *p*-value ≤ 0.01 were considered significant.

## Figures and Tables

**Table 1:** Summary of tissue-specific differentially methylated CpG sites (tDMS).

Tissue	Total	Hypermethylated	Hypomethylated
Sperm	9039	7609	1430
Lymph Node	1359	839	520
Hair	9227	1323	7904
Ear Punch	1300	247	1053
Duodenum	1761	365	1396
Blood	2305	1758	547
Abomasum	1956	434	1522

## Data Availability

The data are contained within the article or Supplementary Materials.

## Supplementary Materials

The following supporting information can be downloaded at: https://www.mdpi.com/article/10.3390/ijms26094284/s1.
